# Finerenone after FINEARTS-HF: evidence boundaries and implementation in heart failure with LVEF ≥40%

**DOI:** 10.3389/fcvm.2026.1880045

**Published:** 2026-06-16

**Authors:** Shihang Feng, Hongguang Jin

**Affiliations:** 1 College of Traditional Chinese Medicine, Changchun University of Chinese Medicine, Changchun, China; 2 The Affiliated Hospital to Changchun University of Chinese Medicine, Changchun, China

**Keywords:** albuminuria, cardiovascular-kidney-metabolic syndrome, finerenone, heart failure, implementation, nonsteroidal mineralocorticoid receptor antagonism

## Abstract

FINEARTS-HF moved finerenone from cardiorenal biological plausibility to randomized outcome evidence for selected adults with symptomatic heart failure (HF) and left ventricular ejection fraction (LVEF) ≥40%. Finerenone reduced the prespecified composite of cardiovascular death and total worsening HF events, with benefit driven mainly by fewer worsening HF events; cardiovascular death as an individual endpoint was not significantly reduced. This focused narrative Review defines the evidence boundaries after FINEARTS-HF and translates them into clinical implementation for cardiovascular-kidney-metabolic (CKM)-burdened HF with LVEF ≥40%. CKM features are used as a practical framework for risk organization, multimorbidity assessment, competing symptom mechanisms, and monitoring intensity, not as diagnostic criteria, eligibility criteria, or validated predictors of relative response. Safe use requires confirmed symptomatic HF, exclusion of mimickers, confirmation of the local prescribing information, baseline potassium and eGFR assessment, HF-specific dosing, interaction review, laboratory reassessment around 4 weeks after initiation and dose changes, and reassessment during acute illness or unstable renal function. Finerenone should complement SGLT2 inhibitors, decongestion, obesity-directed therapy when indicated, atrial fibrillation care, blood-pressure control, exercise, and nutrition. Future studies should quantify absolute benefit across CKM risk, test UACR, biomarker, and imaging mediation, define combination and sequencing strategies, compare safety with steroidal mineralocorticoid receptor antagonists, and evaluate real-world implementation.

## Highlights

- Finerenone reduced the FINEARTS-HF prespecified composite of cardiovascular death and total worsening HF events in eligible patients with symptomatic HF and LVEF ≥40%.
- The observed benefit was driven mainly by fewer worsening HF events. Cardiovascular death as an individual endpoint was not significantly reduced.
- CKM features organize risk, multimorbidity burden, competing symptom mechanisms, and monitoring needs; they are not diagnostic criteria, eligibility criteria, or validated relative-response modifiers.
- Implementation requires confirmed HF, mimicker exclusion, current regional label confirmation, baseline potassium and eGFR assessment, HF-specific dosing, interaction review, and early laboratory follow-up.
- The next research phase should quantify absolute benefit, test biomarker and imaging mediation, define combination strategies, compare safety with steroidal MRAs, and evaluate implementation safety.

## 1 Introduction: the post-FINEARTS-HF translation problem

Heart failure (HF) with left ventricular ejection fraction (LVEF) ≥40% is a heterogeneous clinical syndrome rather than a single disease. It includes HF with mildly reduced ejection fraction (HFmrEF) and HF with preserved ejection fraction (HFpEF), and varies by age, sex, adiposity, kidney function, atrial rhythm, pulmonary vascular reserve, skeletal-muscle function, inflammatory burden, and diagnostic certainty (1–3).

Many patients with HF with LVEF ≥40% carry cardiovascular-kidney-metabolic (CKM) features such as obesity or visceral adiposity, type 2 diabetes (T2D), chronic kidney disease (CKD), albuminuria, hypertension, atrial fibrillation (AF), sleep-disordered breathing, recurrent congestion, and systemic inflammation (4–9). The CKM construct is useful because it describes multiorgan biology and baseline risk. In this Review, CKM features are used to organize risk, multimorbidity burden, competing symptom mechanisms, monitoring needs, and future research hypotheses. They are not used as HF diagnostic criteria, finerenone eligibility criteria, or validated predictors of relative treatment response.

Terminology remains regional and dynamic. The 2025 Canadian Cardiovascular Society/Canadian Heart Failure Society guideline uses heart failure with nonreduced ejection fraction (HFnrEF), defined as signs and symptoms of HF with LVEF >40%, to provide a unified pharmacologic framework (10). Throughout this Review, HF with LVEF ≥40% refers to the FINEARTS-HF trial population and the current label-based threshold in relevant jurisdictions. When discussing Canadian HFnrEF terminology, we preserve the guideline definition of LVEF >40%.

FINEARTS-HF changed the status of finerenone in HF with LVEF ≥40% (11–13). The practical question is no longer only whether the trial was positive. The more important questions are what the trial proved, what remains untested, and how clinicians can implement treatment safely in patients with high CKM burden.

This Review differs from existing summaries in three ways. First, it separates trial-proven outcome evidence from regulatory wording, mechanistic inference, and subgroup hypothesis generation. Second, it translates FINEARTS-HF into a practical implementation and monitoring framework focused on confirmed HF, mimicker exclusion, potassium and eGFR safeguards, HF-specific dosing, and acute-illness reassessment. Third, it uses CKM biology as a risk-organization construct and research framework, not as a new HF entity or validated finerenone-response phenotype. Claims that exceed the evidence, including cardiovascular-death-only benefit, rescue decongestion, validated CKM-response selection, and proven mechanistic mediation, are handled explicitly in the evidence-boundary table rather than as separate boxed material.

## 2 Focused narrative review methods

This is a focused narrative Review, not a systematic review or meta-analysis. The Review was built around three questions: what FINEARTS-HF proved and did not prove; how CKM and mineralocorticoid receptor (MR) biology can be interpreted without overclaiming mediation; and how finerenone can be implemented safely in clinical practice.

We searched PubMed/MEDLINE, Web of Science, ClinicalTrials.gov, major cardiology and HF guideline documents, regulatory documents, and major cardiology/HF congress publications. The search was last updated on 10 May 2026. Search terms included finerenone, FINEARTS-HF, heart failure with preserved ejection fraction, heart failure with mildly reduced ejection fraction, mineralocorticoid receptor antagonist, cardiovascular-kidney-metabolic syndrome, albuminuria, chronic kidney disease, SGLT2 inhibitor, obesity, HFpEF, and HFmrEF.

Evidence priority was assigned to randomized trials, prespecified or regulatory analyses, official prescribing information, major guideline documents, and high-quality human or translational mechanistic studies. No formal duplicate screening, risk-of-bias scoring, PRISMA flow reporting, GRADE assessment, or quantitative pooling was performed. Evidence statements are framed as outcome-supported, clinically plausible, or hypothesis-generating.

## 3 Part I. Evidence boundaries after FINEARTS-HF

### 3.1 What FINEARTS-HF proved

The steroidal mineralocorticoid receptor antagonist (MRA) experience in HFpEF was informative but incomplete. In TOPCAT, spironolactone did not significantly reduce the primary composite endpoint in the overall population, although HF hospitalization was lower and major regional differences raised concerns about selection, adherence, event rates, and drug exposure (14, 15). TOPCAT is best interpreted not as a rejection of MR antagonism in HF with LVEF ≥40%, but as a warning that phenotype ascertainment, adherence, background risk, and pharmacologic exposure determine whether an MR hypothesis is testable.

Finerenone first established a cardiorenal outcome role in CKD associated with T2D. FIDELIO-DKD and FIGARO-DKD reduced kidney and cardiovascular events, and FIDELITY analyses supported biological compatibility with SGLT2 inhibitor use (16–18). These data created a cardiorenal rationale, but they did not prove efficacy in HF with LVEF ≥40%. A dedicated HF outcome trial was required.

FINEARTS-HF enrolled 6,001 patients with symptomatic HF, NYHA class II-IV, LVEF ≥40%, structural heart disease, and elevated natriuretic peptides. Patients were required to have eGFR ≥25 mL/min/1.73 m^2^ and serum potassium ≤5.0 mmol/L at screening and randomization, and most were receiving background HF treatment, including diuretics (12, 13). The trial population was clinically recognizable: mean age 72 years, 46% female, mean LVEF 53%, 64% with LVEF ≥50%, 38% with AF, 41% with diabetes, 48% with eGFR <60 mL/min/1.73 m^2^, and approximately 14% on an SGLT2 inhibitor at baseline (13).

The HFmrEF component is therefore directly represented. Because 64% of participants had LVEF ≥50%, approximately 36% had LVEF 40%–49%. FINEARTS-HF thus supports use in a population that included a substantial HFmrEF stratum, but it does not establish a separate cardiovascular-mortality benefit in HFmrEF or validate LVEF 40%–49% as a distinct relative-response phenotype (12, 13).

Finerenone reduced the prespecified composite of cardiovascular death and total worsening HF events compared with placebo. The US prescribing information reports 1083 events among finerenone-treated patients and 1,283 among placebo-treated patients, corresponding to event rates of 14.9 and 17.7 per 100 patient-years and a rate ratio of 0.84 (95% CI 0.74–0.95; *P* = 0.007) (13). The observed benefit was driven mainly by fewer total worsening HF events, including HF hospitalizations and urgent HF visits (rate ratio 0.82; 95% CI 0.71–0.94; *P* = 0.006; event rates 11.6 vs. 14.1 per 100 patient-years). Cardiovascular death as an individual endpoint was not significantly reduced (hazard ratio 0.93; 95% CI 0.78–1.11; event rates 3.3 vs. 3.6 per 100 patient-years) (12, 13).

This pattern is clinically important. Recurrent HF hospitalizations and urgent HF visits are major patient-centered burdens in HF with LVEF ≥40%, and recurrent-event endpoints better reflect the clinical course than mortality alone. The language must remain precise: finerenone reduced the prespecified composite of cardiovascular death and total worsening HF events in eligible patients with symptomatic HF and LVEF ≥40%; the observed benefit was driven mainly by fewer worsening HF events; cardiovascular death as an individual endpoint was not significantly reduced.

Safety interpretation requires the same discipline. In FINEARTS-HF, finerenone increased potassium elevations at clinically meaningful thresholds while reducing hypokalemia. A dedicated potassium analysis reported a greater risk of serum potassium increasing to >5.5 mmol/L with finerenone than placebo (HR 2.16, 95% CI 1.83–2.56; *P* < 0.001) and a lower risk of serum potassium decreasing to <3.5 mmol/L (HR 0.46, 95% CI 0.38–0.56; *P* < 0.001) (66). Hyperkalemia- and hypokalemia-related hospitalizations were infrequent (hyperkalemia hospitalization 0.5% vs. 0.2%; hypokalemia hospitalization 0.1% vs. 0.2%), and no potassium-related deaths occurred in either group (66). Overall discontinuation of trial drug for reasons other than death was similar between groups (20.4% vs. 20.6%) (12), but renal-function-related adverse reactions still require implementation attention. The US prescribing information reports adverse reactions related to worsening renal function more frequently with finerenone than placebo in FINEARTS-HF (18% vs. 12%), including renal impairment (7% vs. 4%), eGFR decrease (5% vs. 4%), acute kidney injury (4% vs. 2%), and renal failure (3% vs. 2%); most events were mild to moderate, but they led to dose modifications in 9% vs. 4% and hospitalization in 2.0% vs. 1.3% (13). These quantified trade-offs support protocolized potassium and eGFR monitoring, dose adjustment or temporary interruption when thresholds are crossed, and reassessment during unstable physiology rather than indiscriminate avoidance in CKM-burdened patients.

### 3.2 What FINEARTS-HF did not prove

FINEARTS-HF did not prove that finerenone reduces cardiovascular death as a stand-alone endpoint. It did not establish finerenone as rescue decongestion, and it does not replace loop diuretics for active volume overload. It did not prove that CKM burden, obesity, diabetes, albuminuria, or kidney-risk category is a validated relative-response modifier. It did not establish that outcome benefit is mediated by reversal of fibrosis, endothelial dysfunction, mitochondrial dysfunction, or inflammasome activation. It also did not provide definitive factorial proof that finerenone adds outcome benefit to an SGLT2 inhibitor in patients already treated with contemporary background therapy.

Regulatory wording requires care because the United States and European Union indications differ substantively in wording. In the United States, finerenone is indicated to reduce the risk of cardiovascular death, HF hospitalization, and urgent HF visits in adults with HF and LVEF ≥40% (13). In the European Union, EMA regulatory materials describe finerenone as indicated for the treatment of symptomatic chronic HF with LVEF ≥40% in adults (19). These statements reflect regional regulatory decisions and should be reproduced according to the local product information. They do not change the trial-level fact that cardiovascular death alone was not significantly reduced in FINEARTS-HF. Local prescribing information needs rechecking immediately before clinical use, manuscript submission, and proof correction.

Secondary analyses refine implementation but do not replace the primary evidence. Finerenone reduced urine albumin-to-creatinine ratio (UACR) early and persistently and lowered new-onset microalbuminuria and macroalbuminuria, but it did not significantly reduce a low-frequency kidney composite in FINEARTS-HF (20). Kidney-risk analyses suggest consistent relative benefit across kidney-risk categories and greater absolute risk in higher-risk groups; this supports eGFR and UACR as useful phenotype and monitoring markers, but it does not validate albuminuria as a relative treatment-effect modifier (21). Prespecified analyses by glycemic status, obesity, and improved EF support broad consistency of treatment effect, not a CKM-response algorithm (22–25).

A prespecified FINEARTS-HF analysis by SGLT2 inhibitor use found no clear treatment-effect heterogeneity. Baseline SGLT2 inhibitor use was modest, approximately 14%, and time-updated analyses accounting for initiation during follow-up did not meaningfully change the estimate (26). These data support practical co-use when both classes are independently appropriate and safety monitoring is feasible. They do not prove additive efficacy, and they do not define a randomized evidence-based sequencing algorithm.

Generalizability also has boundaries. The evidence applies best to trial-like and label-eligible patients with confirmed symptomatic HF, LVEF ≥40%, potassium and eGFR values within permitted ranges, and feasible laboratory follow-up. Routine extrapolation is not warranted for patients with eGFR <25 mL/min/1.73 m^2^, baseline hyperkalemia above label thresholds, unstable acute illness, active acute kidney injury, severe dehydration, rapidly changing diuretic requirements, or no realistic access to follow-up laboratory testing.

Table 1 consolidates the landmark evidence base, clinically usable interpretation, and boundaries that should not be crossed when translating FINEARTS-HF into practice.

**Table 1:** Landmark evidence and evidence boundaries for finerenone in HF with LVEF ≥40%.

Domain/source	Evidence-supported statement	Clinical translation	Boundary to preserve
TOPCAT and steroidal MRA context	In HFpEF with LVEF ≥45%, spironolactone did not significantly reduce the primary composite overall, although HF hospitalization was lower and regional/exposure concerns were important (14, 15).	MR biology remained testable, but phenotype ascertainment, adherence, background risk, and exposure determine interpretability.	TOPCAT is not a finerenone trial and does not establish head-to-head superiority or inferiority vs. nonsteroidal MR antagonism.
FIDELIO-DKD, FIGARO-DKD, and FIDELITY	Finerenone reduced kidney and cardiovascular events in CKD associated with T2D and supported biological compatibility with SGLT2 inhibitor use (16–18).	These trials provide a cardiorenal rationale for studying finerenone in CKM-burdened HF.	They are not dedicated efficacy evidence for symptomatic HF with LVEF ≥40%.
FINEARTS-HF primary evidence	In symptomatic HF with LVEF ≥40%, structural disease, elevated natriuretic peptides, eGFR ≥25 mL/min/1.73 m², and potassium ≤5.0 mmol/L, finerenone reduced the prespecified composite of cardiovascular death and total worsening HF events (RR 0.84, 95% CI 0.74–0.95) (12, 13).	Outcome evidence applies best to trial-like, label-eligible patients with feasible potassium and eGFR monitoring.	The observed benefit was driven mainly by fewer worsening HF events; cardiovascular death as an individual endpoint was not significantly reduced.
Secondary FINEARTS-HF analyses	UACR fell early and persistently; kidney-risk, glycemic-status, obesity, and improved-EF analyses generally support consistency of clinical effect (20–25).	eGFR and UACR help organize phenotype, baseline risk, monitoring intensity, and absolute-benefit discussions.	CKM burden, obesity, diabetes, albuminuria, and kidney-risk category are not validated relative-response modifiers.
SGLT2 inhibitor context	Prespecified and time-updated analyses showed no clear treatment-effect heterogeneity by SGLT2 inhibitor use, but baseline SGLT2 inhibitor exposure was modest (26).	Co-use can be considered when each class is independently appropriate and potassium/eGFR monitoring is feasible; sequencing should be presented as expert implementation judgment.	These data do not prove additive efficacy and do not define a randomized evidence-based sequencing algorithm.
Mechanistic interpretation and generalizability	MR activation plausibly links CKM stress to sodium avidity, albuminuria, inflammation, oxidative stress, endothelial dysfunction, and fibrosis (31–38).	Mechanistic biology supports research design, monitoring awareness, and a coherent therapeutic rationale.	FINEARTS-HF did not prove mediation through fibrosis reversal, endothelial-mitochondrial pathways, inflammasome signaling, or body-composition change; extrapolation is not warranted to patients outside trial-like or label-eligible ranges, including eGFR <25 mL/min/1.73 m^2^, baseline hyperkalemia above label thresholds, unstable renal function, acute illness, or infeasible laboratory follow-up.
Regulatory wording	US labeling specifies risk reduction for cardiovascular death, HF hospitalization, and urgent HF visits, whereas EU product information frames finerenone as treatment of symptomatic chronic HF with LVEF ≥40% in adults (13, 19).	Use region-specific wording for indication, contraindications, dosing, monitoring, interactions, and reimbursement immediately before use and proof correction.	Regulatory wording does not change the trial-level fact that cardiovascular death alone was not significantly reduced in FINEARTS-HF.

### 3.3 CKM-MR biology: plausible rationale, not proven mediation

The biological rationale for finerenone rests on convergence between CKM stress and MR signaling. MR activation links CKM stress to renal sodium avidity, potassium vulnerability, albuminuria, inflammation, oxidative stress, endothelial dysfunction, tubulointerstitial injury, fibroblast signaling, and myocardial fibrosis (31–38). These pathways connect congestion, kidney dysfunction, metabolic disease, vascular inflammation, and tissue remodeling.

Finerenone has plausible pharmacologic differences from spironolactone and eplerenone as a nonsteroidal MRA. These include differences in molecular structure, receptor interaction, tissue distribution, downstream transcriptional effects, and sex-hormone receptor cross-reactivity (38). Such differences support a dedicated trial rationale. They do not establish head-to-head superiority over steroidal MRAs in HF with LVEF ≥40%.

CKM burden is broader than obesity-HFpEF. Some patients have severe visceral adiposity, insulin resistance, sleep-disordered breathing, and systemic inflammation. Others, especially older adults and some Asian populations, may have modest body mass index but substantial visceral adiposity, sarcopenia, myosteatosis, CKD, AF, or inflammatory aging (39). Skeletal-muscle mitochondrial abnormalities, impaired oxidative capacity, and body-composition changes also contribute to exercise intolerance and symptom burden in HFpEF (47–49). UACR, eGFR, body composition, AF burden, natriuretic peptides interpreted in context, inflammatory biomarkers, functional capacity, and frailty may therefore describe risk and monitoring needs better than body mass index alone.

The evidentiary strength is not uniform. Human HFpEF data most strongly support endothelial inflammation, coronary microvascular dysfunction, myocardial stiffness and extracellular-matrix turnover, skeletal-muscle energetic impairment, sarcopenia or myosteatosis, and body-composition abnormalities as contributors to symptoms, functional limitation, and prognosis (31–37, 47–49, 62, 63). By contrast, mitochondrial danger signaling, TLR9, cGAS-STING, and NLRP3 inflammasome activation remain primarily preclinical or translational pathways in this setting (40–46).

**Figure 1:**
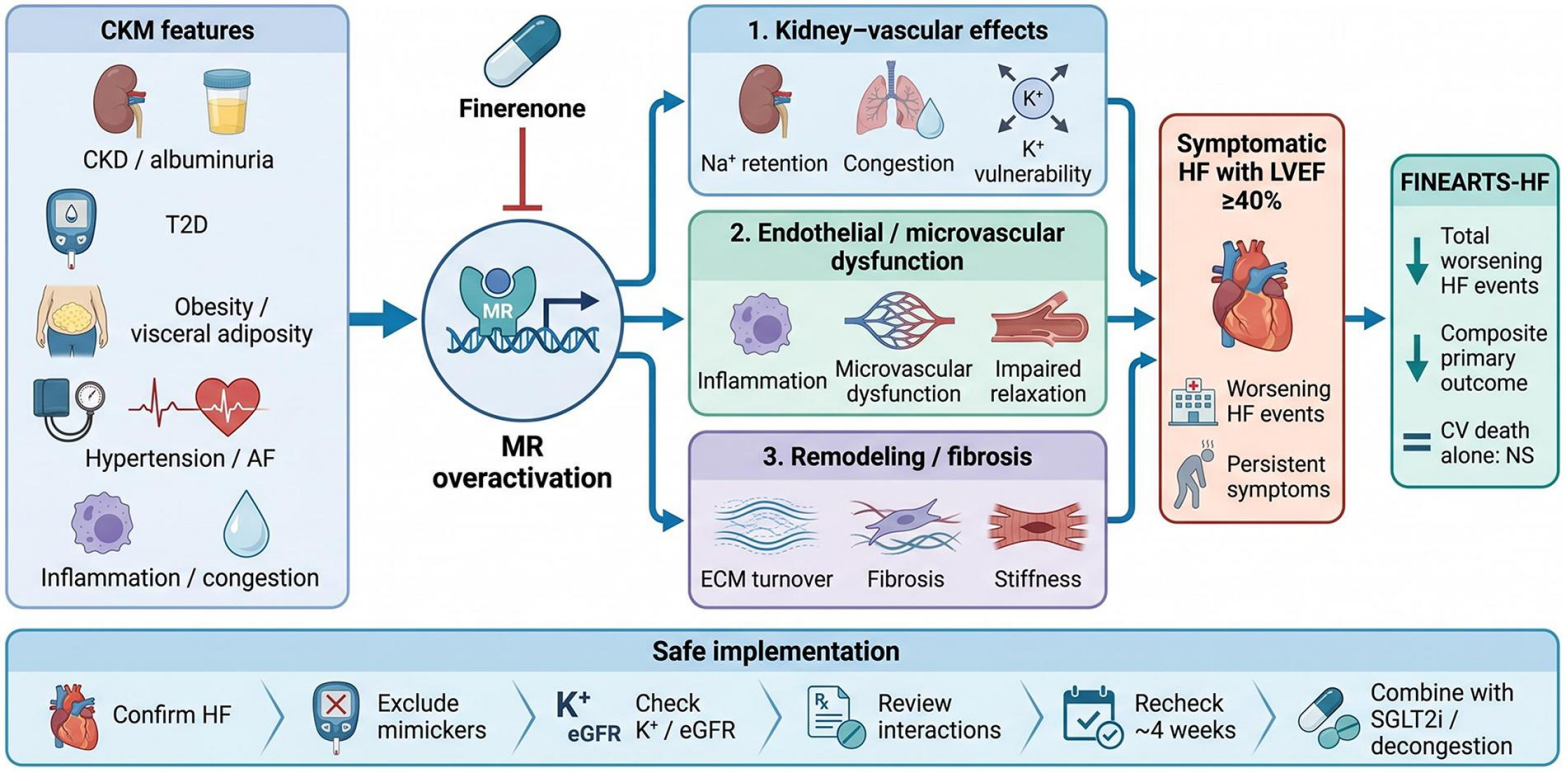
CKM burden, MR overactivation, and finerenone action in heart failure with LVEF ≥40%. CKM features such as CKD, albuminuria, T2D, obesity or visceral adiposity, hypertension, AF, inflammation, and recurrent congestion may promote MR overactivation in HF with LVEF ≥40%. MR overactivation links CKM stress to kidney–vascular effects, endothelial and microvascular dysfunction, and remodeling or fibrosis, contributing to sodium retention, congestion, potassium vulnerability, inflammation, impaired relaxation, extracellular-matrix turnover, fibrosis, stiffness, worsening HF events, and persistent symptoms. Finerenone antagonizes MR signaling and, in FINEARTS-HF, reduced the prespecified composite of cardiovascular death and total worsening HF events, mainly by reducing worsening HF events; cardiovascular death alone was not significantly reduced. Safe implementation requires confirmation of HF, exclusion of mimickers, baseline potassium and eGFR assessment, interaction review, laboratory reassessment around 4 weeks, and integration with SGLT2 inhibitors, decongestion, and comprehensive HF care. CKM features organize risk and monitoring intensity but should not be treated as diagnostic criteria, eligibility criteria, or validated relative-response modifiers.

Finerenone-specific human evidence in HF with LVEF ≥40% currently consists mainly of clinical outcomes, UACR change, potassium/eGFR effects, and regulatory safety data (12, 13, 20, 66). FINEARTS-HF did not prove that clinical benefit is mediated by reversal of endothelial-mitochondrial stress, inflammasome activity, myocardial fibrosis, or body-composition change. CKM-MR biology can guide risk organization, monitoring intensity, and prospective mechanistic research, but it does not yet support routine selection of “finerenone responders.” A compressed evidence-tier framework is provided in Supplementary Table S1 and Figure 1.

## 4 Part II. Clinical implementation in CKM-burdened HF

### 4.1 Confirm HF and exclude mimickers before treatment

Implementation begins with diagnostic discipline. Dyspnea, edema, exertional intolerance, and LVEF ≥40% do not automatically establish HF. Finerenone is best considered only after symptomatic HF has been confirmed and current regional label eligibility has been checked. It is not a diagnostic shortcut in unexplained dyspnea or edema. In uncertain cases, diagnostic clarification takes priority over a therapeutic trial.

Objective evidence may include structural heart disease, abnormal diastolic or filling-pressure indices, pulmonary hypertension attributable to left-sided disease, elevated natriuretic peptides interpreted in context, prior adjudicated HF hospitalization or urgent HF visit, or invasive or exercise evidence of elevated filling pressures. Scores such as H2FPEF and HFA-PEFF, echocardiography, cardiac magnetic resonance imaging, cardiopulmonary exercise testing, exercise echocardiography, or invasive exercise hemodynamics may be needed when diagnostic certainty is low (27–29). These tools help establish the HF syndrome; they are not finerenone eligibility criteria.

Natriuretic peptides require bidirectional interpretation. Obesity can suppress BNP and NT-proBNP, whereas AF, older age, and kidney dysfunction may raise concentrations independent of acute congestion. Discordance between symptoms, natriuretic peptides, structural findings, and volume status warrants further assessment before therapy is assigned to HF.

Active search for mimickers and dominant contributors is essential. Important alternatives include transthyretin cardiac amyloidosis, hypertrophic cardiomyopathy, constrictive pericarditis, occult valvular disease, coronary ischemia, pulmonary venous or pulmonary arterial disease, obesity hypoventilation, severe anemia, thyroid disease, high-output states, iatrogenic fluid overload, medication-related edema, and primary renal fluid retention. Amyloidosis is particularly important in older adults with disproportionate wall thickness, discordant low electrocardiographic voltage, carpal tunnel syndrome, neuropathy, or progressive unexplained HF because disease-modifying treatment is available (30).

CKM features then need documentation because they organize risk and monitoring needs: CKD, UACR ≥30 mg/g, diabetes or insulin resistance, visceral adiposity, resistant hypertension, AF, sleep-disordered breathing, systemic inflammation, frailty, sarcopenic adiposity, and recurrent congestion. Higher CKM burden may identify higher baseline risk and therefore greater potential absolute benefit, even when relative treatment effects are consistent. This distinction avoids treating risk enrichment as proof of response modification.

### 4.2 Label-consistent initiation, monitoring, and interruption safeguards

Finerenone implementation is best framed as a safeguard-based process, not as a substitute for prescribing information. Local prescribing information needs review before use. The operational questions are straightforward: does the patient have confirmed symptomatic HF with LVEF ≥40%; does the patient meet the current regional indication; do serum potassium, eGFR, concomitant drugs, and follow-up capacity allow safe initiation; and is finerenone being added for chronic risk reduction rather than rescue decongestion?.

Measure serum potassium and eGFR before initiation. Do not initiate treatment when potassium or renal function falls outside current local label thresholds. Serum potassium is reported in mmol/L in this Review; US prescribing information uses mEq/L, which is numerically equivalent for potassium. In the US label, Kerendia should not be initiated if serum potassium is >5.0 mEq/L, and initiation in HF with eGFR <25 mL/min/1.73 m² is not recommended (13). This is a jurisdiction-specific example, not a universal prescribing instruction.

HF-specific dosing must not be confused with CKD-T2D dosing. In the US label, the recommended initial dose is based on eGFR: 20 mg once daily when eGFR is ≥60 mL/min/1.73 m^2^ and 10 mg once daily when eGFR is ≥25 to <60 mL/min/1.73 m^2^. The HF target daily dose is 40 mg once daily if eGFR at initiation is ≥60 mL/min/1.73 m^2^ and 20 mg once daily if eGFR at initiation is ≥25 to <60 mL/min/1.73 m^2^, with subsequent adjustment by potassium and eGFR (13). The CKD-T2D target dose must not be imported into HF implementation.

Medication review is mandatory. Strong CYP3A4 inhibitors are contraindicated in the US label; grapefruit intake should be avoided; moderate or weak CYP3A4 inhibitors warrant potassium monitoring and dose consideration; and strong or moderate CYP3A4 inducers are generally avoided because they may reduce exposure (13). The review also includes potassium supplements, potassium-containing salt substitutes, nonsteroidal anti-inflammatory drugs, trimethoprim, renin-angiotensin-aldosterone system inhibitors, heparin exposure, dehydration risk, recent or planned diuretic changes, and other drugs that impair potassium excretion or increase serum potassium.

Reassess potassium and eGFR around 4 weeks after initiation and around 4 weeks after each dose adjustment, with periodic monitoring thereafter (13, 19). More frequent checks are appropriate in CKD, frailty, older age, polypharmacy, recurrent hyperkalemia, high-normal baseline potassium, recent acute kidney injury, major loop-diuretic changes, intercurrent illness, or limited access to urgent laboratory testing.

Acute illness warrants reassessment. Active acute kidney injury, dehydration, severe infection, hospitalization, unstable renal function, rapidly changing diuretic requirements, poor oral intake, or recurrent hyperkalemia may require delay, temporary interruption, repeat laboratory testing, or cautious re-initiation after stabilization. Automatic continuation through unstable physiology is inappropriate simply because finerenone is intended as chronic risk-reduction therapy.

**Figure 2:**
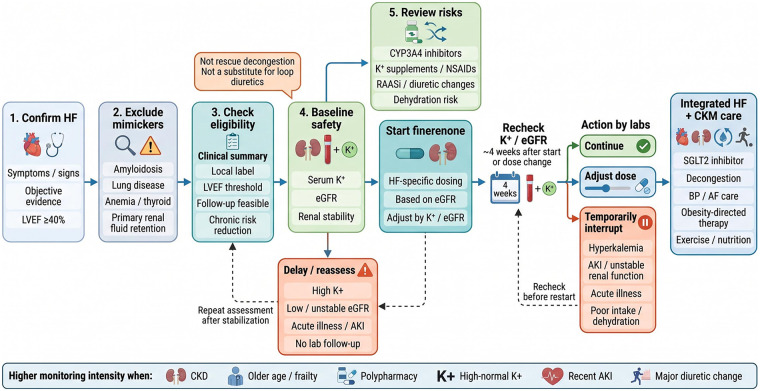
Practical implementation of finerenone in heart failure with LVEF ≥40%. Finerenone implementation in HF with LVEF ≥40% should follow a safeguard-based sequence: confirm symptomatic HF with objective evidence and LVEF ≥40%, exclude mimickers or dominant alternative causes of symptoms, verify local label eligibility and follow-up feasibility, and assess baseline serum potassium, eGFR, and renal stability. Finerenone should be used for chronic risk reduction, not rescue decongestion or replacement of loop diuretics. Initiation should be delayed or reassessed in patients with high potassium, low or unstable eGFR, acute illness or acute kidney injury, or no feasible laboratory follow-up. Before treatment, clinicians should review CYP3A4 inhibitors, potassium supplements, NSAIDs, RAAS inhibitors, diuretic changes, and dehydration risk. HF-specific dosing should be based on eGFR and adjusted according to potassium and eGFR. Laboratory testing should be repeated around 4 weeks after initiation or dose change, with continuation, dose adjustment, or temporary interruption guided by potassium, renal function, acute illness, intake, and hydration status. Higher monitoring intensity is warranted in CKD, older age or frailty, polypharmacy, high-normal potassium, recent AKI, and major diuretic changes. Finerenone should be integrated with SGLT2 inhibitors, decongestion, blood-pressure and AF care, obesity-directed therapy when indicated, exercise, and nutrition.

Table 2 translates these principles into operational safeguards, and Figure 2 shows the corresponding workflow from diagnostic confirmation to integrated chronic HF care.

**Table 2:** Practical safeguards before and after finerenone initiation in HF with LVEF ≥40% .

Domain	Operational safeguard	Practical implication
Confirm HF	Symptoms/signs compatible with HF plus objective evidence such as structural/functional disease, elevated filling pressures, natriuretic peptides interpreted in context, or prior adjudicated HF events.	Do not use finerenone as a diagnostic shortcut in unexplained dyspnea, edema, lung disease, obesity hypoventilation, anemia, or primary renal fluid retention.
Confirm LVEF and terminology	Use HF with LVEF ≥40% for FINEARTS-HF and label-based discussion; preserve regional definitions such as HFnrEF >40% when discussing Canadian guidance.	Avoid substituting guideline terminology for trial or label eligibility at the 40% threshold.
Check local prescribing information	Review the current indication, contraindications, dosing table, monitoring instructions, interactions, and reimbursement criteria before use.	Label wording differs by region and may change between submission and publication.
Assess safe initiation	Measure serum potassium and eGFR; confirm follow-up capacity.	Defer, avoid, or reassess therapy when potassium or eGFR fall outside label thresholds, renal function is unstable, or monitoring is infeasible.
Use HF-specific dose target	Use the HF indication dosing pathway; in the US label, initial and target doses are eGFR dependent.	Do not import the CKD-T2D target dose into HF care.
Review interactions and potassium risk	Review strong CYP3A4 inhibitors, CYP3A4 inducers, grapefruit, potassium supplements or salt substitutes, NSAIDs, trimethoprim, RAAS inhibitors, heparin exposure, dehydration risk, and diuretic changes.	Modify interacting drugs where possible and increase monitoring when potassium risk is high.
Recheck laboratories	Reassess potassium and eGFR around 4 weeks after initiation and after each dose adjustment; monitor periodically thereafter.	Use more frequent checks in CKD, frailty, older age, polypharmacy, recurrent hyperkalemia, high-normal baseline potassium, recent AKI, major loop-diuretic changes, intercurrent illness, or limited lab access.
Respond to acute illness	AKI, dehydration, severe infection, hospitalization, unstable renal function, poor oral intake, or recurrent hyperkalemia triggers reassessment.	Temporary interruption and repeat labs may be safer than automatic continuation during unstable physiology.

### 4.3 Integration with SGLT2 inhibitors, obesity-directed therapy, and standard HF care

Finerenone complements, rather than competes with, SGLT2 inhibitors. EMPEROR-Preserved and DELIVER established empagliflozin and dapagliflozin as foundational therapies across much of the HFmrEF/HFpEF spectrum, with the most consistent benefit being fewer worsening HF events and HF hospitalizations (50–52). SGLT2 inhibitors remain foundational for most eligible patients with HFmrEF/HFpEF or HF with LVEF ≥40%, with strength of recommendation varying by region and publication date (53, 54).

In practice, SGLT2 inhibitors are usually considered early for eligible patients with HF and LVEF ≥40%, together with decongestion and blood-pressure optimization, consistent with contemporary guideline-based HF care (53, 54). Finerenone may be added or sequenced according to regional label eligibility, kidney function, potassium, CKM risk, access, patient preference, and monitoring feasibility. This sequencing language reflects expert implementation judgment rather than direct randomized sequencing evidence. FINEARTS-HF subgroup and time-updated analyses found no clear treatment-effect heterogeneity by SGLT2 inhibitor use, but baseline SGLT2 inhibitor exposure was modest and the trial was not designed as a factorial test of additive efficacy (26).

Obesity-directed incretin therapy is complementary management for obesity-related HFpEF, not a direct substitute for or comparator with finerenone. Semaglutide 2.4 mg improved symptoms, physical limitations, body weight, 6 min walk distance, and inflammation in STEP-HFpEF and STEP-HFpEF DM, but these were primarily symptom and function trials rather than hard-outcome trials (55, 56). In SUMMIT, tirzepatide reduced the composite of cardiovascular death or worsening HF events in HFpEF with obesity. The event reduction was driven mainly by fewer worsening HF events, and the trial should not be interpreted as definitive evidence for a stand-alone cardiovascular mortality benefit (57). EMA assessment did not recommend a separate HFpEF indication for Mounjaro but noted that relevant study data would be included in product information (58).

Phenotype guides prioritization, but the following examples are illustrative implementation scenarios rather than evidence-derived treatment-selection algorithms. CKM features are used here to organize competing risks, monitoring needs, and co-interventions, not to define finerenone eligibility or predict relative response. A patient with albuminuric CKD, diabetes, recurrent congestion, and LVEF 55% may require SGLT2 inhibition, label-eligible finerenone, loop-diuretic adjustment, blood-pressure control, and kidney-metabolic risk reduction. A patient with severe obesity, low natriuretic peptides, high symptom burden, and preserved kidney function may need obesity-directed therapy, exercise, nutrition, and lean-mass preservation, with finerenone considered only if confirmed HF and MR/cardiorenal risk coexist. A frail non-obese patient with CKD, AF, sarcopenia, and low peak oxygen uptake may need cardiorenal protection, rhythm or rate strategy, resistance training, protein optimization, and careful monitoring more than aggressive weight loss.

Standard HF care remains essential. Treat congestion, hypertension, AF, ischemia, valvular disease, sleep-disordered breathing, iron deficiency, frailty, and rehabilitation needs. Finerenone reduces worsening HF risk in eligible patients, but it does not replace decongestion or individualized management of dominant contributors such as AF, hypertension, ischemia, valvular disease, sleep-disordered breathing, iron deficiency, frailty, physical inactivity, or poor nutrition. The post-hospitalization vulnerable phase also argues for early reassessment of volume status, kidney function, potassium, and persistence with disease-modifying therapy (59).

## 5 Part III. Research agenda

### 5.1 Biomarkers, imaging, and endpoints for finerenone-era studies

Biomarkers are best organized by use case. Clinical-now measures include serum potassium, eGFR, UACR, BNP or NT-proBNP, HbA1c, lipids, and iron indices. Risk-enrichment measures may include cystatin C, high-sensitivity troponin, high-sensitivity C-reactive protein, frailty measures, and body composition. Mechanistic research may use IL-6, TNF-receptor pathways, soluble ST2, GDF-15, galectin-3, extracellular-matrix turnover markers such as PRO-C3, endothelial activation markers, proteomics, metabolomics, and circulating mitochondrial DNA. These tools mainly serve research agendas and selected clinical questions; they are not routine finerenone eligibility tests.

UACR deserves special emphasis. It is inexpensive, widely available, biologically linked to endothelial and kidney injury, and operationally useful for CKM phenotyping. FINEARTS-HF kidney analyses show early and sustained UACR reduction with finerenone and consistent clinical benefit across kidney-risk categories (20, 21). UACR is useful as a risk, phenotype, and pharmacodynamic marker, but mediation of HF benefit remains unproven.

Endpoints need to reflect the lived experience of HF with LVEF ≥40%. Time to first hospitalization is inadequate for a recurrent syndrome. Total worsening HF events, urgent HF visits, outpatient intravenous diuretic treatment, oral diuretic intensification, recurrent-event analyses, Kansas City Cardiomyopathy Questionnaire change, exercise capacity, frailty measures, and quality-adjusted survival deserve more consistent use (60, 61).

Imaging can move beyond resting LVEF when the question requires it. Echocardiography remains first-line for structure, filling pressures, valve disease, and pulmonary pressure estimates. Cardiac magnetic resonance imaging can quantify extracellular volume and diffuse fibrosis, whereas stress perfusion cardiac magnetic resonance imaging or positron-emission tomography can assess myocardial blood-flow reserve and coronary microvascular dysfunction (62, 63). CT or cardiac magnetic resonance imaging can quantify epicardial, pericardial, visceral, and skeletal-muscle compartments; dual-energy x-ray absorptiometry can track lean mass during weight-loss therapy. These tools are most useful for mechanistic substudies and selected clinical questions rather than routine finerenone eligibility.

### 5.2 Research priorities after FINEARTS-HF

The next phase needs to test mechanisms and implementation rather than simply add *post hoc* subgroup labels. Prior HFpEF research agendas emphasized mechanistic phenotyping, endpoint refinement, and pragmatic trial design; FINEARTS-HF now makes those priorities more concrete by providing a trial-supported MR antagonist around which biomarker and imaging studies can be designed (64).

Inflammation-enriched trials illustrate the direction of the field. HERMES is evaluating IL-6 pathway inhibition with ziltivekimab in HF with LVEF >40% and systemic inflammation (65). Its relevance for finerenone is conceptual rather than direct: future trials can enrich for measurable mechanisms, use recurrent-event endpoints, and test whether clinical benefit tracks with pathway change.

Table 3 summarizes the proposed research agenda while preserving the distinction between absolute-risk enrichment, relative-response modification, and mechanistic mediation.

**Table 3:** Research priorities after FINEARTS-HF.

Priority question	Preferred design	Minimum dataset/endpoints	Why it matters
Absolute benefit and safety by CKM risk	Pragmatic trial or registry-based recurrent-event model stratified by eGFR, UACR, diabetes, congestion, frailty, and polypharmacy.	Total worsening HF events, potassium and eGFR events, interruption, discontinuation, quality of life.	Separates high baseline risk and absolute benefit from unproven relative-response modification.
UACR and biomarker mediation	Serial biomarker substudy embedded in an outcomes trial or high-quality cohort.	Baseline, 4-week, and 3- to 6-month UACR; eGFR slope; natriuretic peptides; inflammatory and fibrosis markers; recurrent HF events.	Determines whether UACR is a mediator, pharmacodynamic marker, or risk marker.
Microvascular and fibrosis reversibility	CMR/PET mechanistic substudy with prespecified imaging endpoints.	Extracellular volume, myocardial blood-flow reserve, extracellular-matrix biomarkers, KCCQ, exercise capacity.	Tests whether MR antagonism modifies structural or microvascular biology in humans.
Combination and sequencing strategy	Factorial, adaptive, or platform trial of SGLT2 inhibitor, finerenone, and obesity-directed therapy where appropriate.	Total worsening HF events, potassium/eGFR safety, adherence, persistence, cost-effectiveness, patient-reported outcomes.	Guides implementation beyond parallel class recommendations.
Comparative safety and real-world implementation	Pragmatic randomized comparison or high-quality observational comparative-effectiveness study; health-system implementation registry.	Hyperkalemia, renal-function change, gynecomastia/sex-hormone effects, AKI, monitoring completion, discontinuation, cost, rurality, disparities.	Determines whether trial benefit can be translated safely to patients with high absolute risk and variable monitoring access.

### 5.3 Limitations of this review

This Review is a narrative synthesis rather than a systematic review. It does not include formal risk-of-bias assessment, duplicate screening, PRISMA flow reporting, GRADE assessment, or quantitative pooling. Mechanistic evidence is heterogeneous and often derived from preclinical studies, human observational studies, or small mechanistic cohorts rather than randomized mediation analyses.

Regulatory status, prescribing information, reimbursement criteria, and guideline recommendations are changing rapidly and differ by region. This Review was completed during an active period of regulatory updates and secondary analyses; therefore, label wording, reimbursement criteria, and *post hoc* or prespecified analysis details may change between submission and publication. Label-related statements require rechecking immediately before submission and again at proof correction.

Several finerenone-era subgroup and secondary analyses are hypothesis-refining rather than definitive treatment-effect modification studies. Many cited FINEARTS-HF secondary analyses arise from overlapping investigator groups and the same parent trial dataset; this strengthens internal consistency but should not be mistaken for independent replication. Trial inclusion and exclusion criteria, racial and regional representativeness, frailty, polypharmacy, monitoring access, and reimbursement may influence real-world translation.

The research agenda proposed in this Review was framed by the authors and was not developed through formal patient or caregiver involvement. Consequently, patient-prioritized outcomes, monitoring burden, treatment burden, affordability, treatment persistence, and preferences about trade-offs between fewer worsening HF events and additional laboratory surveillance may be incompletely represented.

Finally, the CKM-informed framework proposed here is intended to aid evidence interpretation, implementation, monitoring, and trial design. It does not replace approved indications, local prescribing information, local guidelines, or individualized clinical judgment.

### 5.4 Conclusions

Finerenone has provided dedicated outcome evidence for nonsteroidal MR antagonism in eligible patients with symptomatic HF and LVEF ≥40%. The most accurate post-FINEARTS-HF statement is that finerenone reduced the prespecified composite of cardiovascular death and total worsening HF events, with benefit driven mainly by fewer worsening HF events. Cardiovascular death as an individual endpoint was not significantly reduced.

The most coherent implementation context is CKM-informed care, where CKD, albuminuria, T2D, adiposity, hypertension, AF, inflammation, and recurrent congestion help organize risk and monitoring. CKM burden must not be converted into a new HF diagnosis, a finerenone eligibility criterion, or a validated relative-response phenotype.

The practical message is precision with safeguards: confirm HF rigorously, exclude mimickers, check the local prescribing information, measure potassium and eGFR, review interactions, use HF-specific dosing targets, monitor early, and reassess during acute illness or unstable renal function. Finerenone belongs within integrated care that includes SGLT2 inhibitors, obesity-directed therapy when indicated, decongestion, AF management, blood-pressure control, exercise, and nutrition. The next phase needs to test absolute benefit, biomarker and imaging mediation, combination strategy, comparative safety, and real-world implementation.

## References

[1] Bozkurt B Coats AJS Tsutsui H Abdelhamid CM Adamopoulos S Albert N. Universal definition and classification of heart failure: a report of the heart failure society of America, heart failure association of the European Society of Cardiology, Japanese heart failure society and writing committee of the universal definition of heart failure: endorsed by the Canadian heart failure society, heart failure association of India, cardiac society of Australia and New Zealand, and Chinese heart failure association. Eur J Heart Fail. (2021) 23(3):352–80. 10.1002/ejhf.2115 33605000

[2] Borlaug BA Sharma K Shah SJ Ho JE. Heart failure with preserved ejection fraction: JACC scientific statement. J Am Coll Cardiol. (2023) 81(18):1810–34. 10.1016/j.jacc.2023.01.049 37137592

[3] Shah SJ Katz DH Selvaraj S Burke MA Yancy CW Gheorghiade M. Phenomapping for novel classification of heart failure with preserved ejection fraction. Circulation. (2015) 131(3):269–79. 10.1161/CIRCULATIONAHA.114.010637 25398313 PMC4302027

[4] Ndumele CE Rangaswami J Chow SL Neeland IJ Tuttle KR Khan SS. Cardiovascular-Kidney-Metabolic Health: a presidential advisory from the American Heart Association. Circulation. (2023) 148(20):1606–35. 10.1161/CIR.0000000000001184; Epub 2023 October 9. Erratum in: Circulation. 2024 March 26;149(13):e1023. doi: 10.1161/CIR.0000000000001241. 37807924

[5] Ndumele CE Neeland IJ Tuttle KR Chow SL Mathew RO Khan SS. A synopsis of the evidence for the science and clinical management of cardiovascular-kidney-metabolic (CKM) syndrome: a scientific statement from the American Heart Association. Circulation. (2023) 148(20):1636–64. 10.1161/CIR.0000000000001186 37807920

[6] Packer M Lam CSP Lund LH Maurer MS Borlaug BA. Characterization of the inflammatory-metabolic phenotype of heart failure with a preserved ejection fraction: a hypothesis to explain influence of sex on the evolution and potential treatment of the disease. Eur J Heart Fail. (2020) 22(9):1551–67. 10.1002/ejhf.1902 32441863 PMC7687188

[7] Obokata M Reddy YNV Pislaru SV Melenovsky V Borlaug BA. Evidence supporting the existence of a distinct obese phenotype of heart failure with preserved ejection fraction. Circulation. (2017) 136(1):6–19. 10.1161/CIRCULATIONAHA.116.026807 28381470 PMC5501170

[8] Ostrominski JW Claggett BL Miao ZM Mc Causland FR Anand IS Desai AS. Cardiovascular-Kidney-Metabolic overlap in heart failure with mildly reduced or preserved ejection fraction: a trial-level analysis. J Am Coll Cardiol. (2024) 84(2):223–8. 10.1016/j.jacc.2024.05.005 38744407

[9] Lassen MCH Ostrominski JW Claggett BL Packer M Zile M Desai AS. Cardiovascular-kidney-metabolic overlap in heart failure with preserved ejection fraction: cardiac structure and function, clinical outcomes, and response to sacubitril/valsartan in PARAGON-HF. Eur J Heart Fail. (2024) 26(8):1762–74. 10.1002/ejhf.3304 38932589

[10] Virani S Zieroth S Aleksova N Anderson K Clarke B Ducharme A. Canadian Cardiovascular Society/Canadian heart failure society 2025 guideline update for pharmacologic management of heart failure with nonreduced ejection fraction (LVEF > 40%). Can J Cardiol. (2025) 41(10):1857–74. 10.1016/j.cjca.2025.07.027; Erratum in: Can J Cardiol. 2025 December 23:S0828-282X(25)01558-2. doi: 10.1016/j.cjca.2025.12.001. Erratum in: Can J Cardiol. 2026 January 6:S0828-282X(25)01595-8. doi: 10.1016/j.cjca.2025.12.030. Erratum in: Can J Cardiol. 2026 April 11:S0828-282X(26)00275-8. doi: 10.1016/j.cjca.2026.03.026. 41110921

[11] Halaseh R Sauer AJ Vardeny O Canonico ME Harrington J Svetlichnaya J. A fine addition: finerenone in the evolving landscape of heart failure with preserved ejection fraction. Heart Fail Rev. (2025) 30(2):287–91. 10.1007/s10741-024-10462-2 39476221

[12] Solomon SD McMurray JJV Vaduganathan M Claggett B Jhund PS Desai AS. Finerenone in heart failure with mildly reduced or preserved ejection fraction. N Engl J Med. (2024) 391(16):1475–85. 10.1056/NEJMoa2407107 39225278

[13] Bayer HealthCare Pharmaceuticals Inc. KERENDIA® (finerenone) tablets, for oral use: prescribing information. Whippany, NJ: Bayer HealthCare Pharmaceuticals Inc.; revised July 2025.

[14] Pitt B Pfeffer MA Assmann SF Boineau R Anand IS Claggett B. Spironolactone for heart failure with preserved ejection fraction. N Engl J Med. (2014) 370(15):1383–92. 10.1056/NEJMoa1313731 24716680

[15] Pfeffer MA Claggett B Assmann SF Boineau R Anand IS Clausell N. Regional variation in patients and outcomes in the treatment of preserved cardiac function heart failure with an aldosterone antagonist (TOPCAT) trial. Circulation. (2015) 131(1):34–42. 10.1161/CIRCULATIONAHA.114.013255 25406305

[16] Bakris GL Agarwal R Anker SD Pitt B Ruilope LM Rossing P. FIDELIO-DKD Investigators. Effect of finerenone on chronic kidney disease outcomes in type 2 diabetes. N Engl J Med. (2020) 383(23):2219–29. 10.1056/NEJMoa2025845 33264825

[17] Pitt B Filippatos G Agarwal R Anker SD Bakris GL Rossing P. Cardiovascular events with finerenone in kidney disease and type 2 diabetes. N Engl J Med. (2021) 385(24):2252–63. 10.1056/NEJMoa2110956 34449181

[18] Rossing P Anker SD Filippatos G Pitt B Ruilope LM Birkenfeld AL. Finerenone in patients with chronic kidney disease and type 2 diabetes by sodium-glucose cotransporter 2 inhibitor treatment: the FIDELITY analysis. Diabetes Care. (2022) 45(12):2991–8. 10.2337/dc22-0294 35972218 PMC9862372

[19] European Medicines Agency. Kerendia: EPAR — Product Information. Amsterdam: European Medicines Agency; first published 11 March 2022; Accessed 7 May 2026. DOI: not applicable. Journal: not applicable.

[20] Causland FR M Vaduganathan M Claggett BL Kulac IJ Desai AS Jhund PS. Finerenone and kidney outcomes in patients with heart failure: the FINEARTS-HF trial. J Am Coll Cardiol. (2025) 85(2):159–68. 10.1016/j.jacc.2024.10.091 39490700

[21] Ostrominski JW Mc Causland FR Claggett BL Desai AS Jhund PS Lam CSP. Finerenone across the spectrum of kidney risk in heart failure: the FINEARTS-HF trial. JACC Heart Fail. (2026) 14(1):102439. 10.1016/j.jchf.2025.03.006 40208137

[22] Butt JH Jhund PS Henderson AD Claggett BL Desai AS Lam CSP. Finerenone, glycaemic status, and heart failure with mildly reduced or preserved ejection fraction: a prespecified analysis of the FINEARTS-HF trial. Eur J Heart Fail. (2025) 27(7):1326–41. 10.1002/ejhf.3649 40211489 PMC12370580

[23] Butt JH Henderson AD Jhund PS Claggett BL Desai AS Lay-Flurrie J. Finerenone, obesity, and heart failure with mildly reduced/preserved ejection fraction: prespecified analysis of FINEARTS-HF. J Am Coll Cardiol. (2025) 85(2):140–55. 10.1016/j.jacc.2024.10.111 39665701

[24] Pabon MA Vardeny O Vaduganathan M Desai AS Claggett BL Kulac IJ. Finerenone in heart failure with improved ejection fraction: the FINEARTS-HF randomized clinical trial. JAMA Cardiol. (2025) 10(7):740–5. 10.1001/jamacardio.2025.1101 40397470 PMC12096322

[25] Ostrominski JW Filippatos G Claggett BL Miao ZM Desai AS Jhund PS. Efficacy and safety of finerenone in heart failure with preserved ejection fraction: a FINE-HEART analysis. JACC Heart Fail. (2025) 13(8):102497. 10.1016/j.jchf.2025.03.041 40505158

[26] Vaduganathan M Claggett BL Kulac IJ Miao ZM Desai AS Jhund PS. Effects of the nonsteroidal MRA finerenone with and without concomitant SGLT2 inhibitor use in heart failure. Circulation. (2025) 151(2):149–58. 10.1161/CIRCULATIONAHA.124.072055 39340828 PMC11732259

[27] Kittleson MM Panjrath GS Amancherla K Davis LL Deswal A Dixon DL. 2023 ACC expert consensus decision pathway on management of heart failure with preserved ejection fraction: a report of the American College of Cardiology solution set oversight committee. J Am Coll Cardiol. (2023) 81(18):1835–78. 10.1016/j.jacc.2023.03.393 37137593

[28] Reddy YNV Carter RE Obokata M Redfield MM Borlaug BA. A simple, evidence-based approach to help guide diagnosis of heart failure with preserved ejection fraction. Circulation. (2018) 138(9):861–70. 10.1161/CIRCULATIONAHA.118.034646 29792299 PMC6202181

[29] Pieske B Tschöpe C de Boer RA Fraser AG Anker SD Donal E. How to diagnose heart failure with preserved ejection fraction: the HFA-PEFF diagnostic algorithm: a consensus recommendation from the heart failure association (HFA) of the European Society of Cardiology (ESC). Eur Heart J. (2019) 40(40):3297–317. 10.1093/eurheartj/ehz641; Erratum in: Eur Heart J. 2021 March 31;42(13):1274. doi: 10.1093/eurheartj/ehaa1016. 31504452

[30] Maurer MS Schwartz JH Gundapaneni B Elliott PM Merlini G Waddington-Cruz M. ATTR-ACT Study investigators. Tafamidis treatment for patients with transthyretin amyloid cardiomyopathy. N Engl J Med. (2018) 379(11):1007–16. 10.1056/NEJMoa1805689 30145929

[31] Paulus WJ Tschöpe C. A novel paradigm for heart failure with preserved ejection fraction: comorbidities drive myocardial dysfunction and remodeling through coronary microvascular endothelial inflammation. J Am Coll Cardiol. (2013) 62(4):263–71. 10.1016/j.jacc.2013.02.092 23684677

[32] Franssen C Chen S Unger A Korkmaz HI De Keulenaer GW Tschöpe C. Myocardial microvascular inflammatory endothelial activation in heart failure with preserved ejection fraction. JACC Heart Fail. (2016) 4(4):312–24. 10.1016/j.jchf.2015.10.007 26682792

[33] El Mouhayyar C Chhikara M Tang M Nigwekar SU. Clinical implications of mineralocorticoid receptor overactivation. Clin Kidney J. (2024) 18(1):sfae346. 10.1093/ckj/sfae346; Erratum in: Clin Kidney J. 2025 May 16;18(5):sfaf145. doi: 10.1093/ckj/sfaf145. 39781481 PMC11704795

[34] Cuijpers I Simmonds SJ van Bilsen M Czarnowska E González Miqueo A Heymans S. Microvascular and lymphatic dysfunction in HFpEF and its associated comorbidities. Basic Res Cardiol. (2020) 115(4):39. 10.1007/s00395-020-0798-y 32451732 PMC7248044

[35] Zile MR Baicu CF Ikonomidis JS Stroud RE Nietert PJ Bradshaw AD. Myocardial stiffness in patients with heart failure and a preserved ejection fraction: contributions of collagen and titin. Circulation. (2015) 131(14):1247–59. 10.1161/CIRCULATIONAHA.114.013215 25637629 PMC4390480

[36] Mishra S Kass DA. Cellular and molecular pathobiology of heart failure with preserved ejection fraction. Nat Rev Cardiol. (2021) 18(6):400–23. 10.1038/s41569-020-00480-6; Epub 2021 January 11. Erratum in: Nat Rev Cardiol. 2021 Oct;18(10):735. doi: 10.1038/s41569-021-00516-5. 33432192 PMC8574228

[37] Adamo L Rocha-Resende C Prabhu SD Mann DL. Reappraising the role of inflammation in heart failure. Nat Rev Cardiol. (2020) 17(5):269–85. 10.1038/s41569-019-0315-x; Epub 2020 January 22. Erratum in: Nat Rev Cardiol. 2021 Oct;18(10):735. doi: 10.1038/s41569-021-00534-3. 31969688

[38] Agarwal R Kolkhof P Bakris G Bauersachs J Haller H Wada T. Steroidal and non-steroidal mineralocorticoid receptor antagonists in cardiorenal medicine. Eur Heart J. (2021) 42(2):152–61. 10.1093/eurheartj/ehaa736 33099609 PMC7813624

[39] Seki Y Obokata M Harada T Kagami K Sorimachi H Saito Y. Adiposity and clinical outcomes in east Asian patients with heart failure and preserved ejection fraction. Int J Cardiol Heart Vasc. (2022) 44:101162. 10.1016/j.ijcha.2022.101162 36510581 PMC9735262

[40] Bertero E Maack C. Metabolic remodelling in heart failure. Nat Rev Cardiol. (2018) 15(8):457–70. 10.1038/s41569-018-0044-6 29915254

[41] Cai Z Wu C Xu Y Cai J Zhao M Zu L. The NO-cGMP-PKG axis in HFpEF: from pathological mechanisms to potential therapies. Aging Dis. (2023) 14(1):46–62. 10.14336/AD.2022.0523 36818566 PMC9937694

[42] Lopaschuk GD Karwi QG Tian R Wende AR Abel ED. Cardiac energy metabolism in heart failure. Circ Res. (2021) 128(10):1487–513. 10.1161/CIRCRESAHA.121.318241 33983836 PMC8136750

[43] Schiattarella GG Altamirano F Tong D French KM Villalobos E Kim SY. Nitrosative stress drives heart failure with preserved ejection fraction. Nature. (2019) 568(7752):351–6. 10.1038/s41586-019-1100-z 30971818 PMC6635957

[44] Oka T Hikoso S Yamaguchi O Taneike M Takeda T Tamai T. Mitochondrial DNA that escapes from autophagy causes inflammation and heart failure. Nature. (2012) 485(7397):251–5. 10.1038/nature10992; Erratum in: Nature. 2012 October 11;490(7419):292. 22535248 PMC3378041

[45] Nakayama H Otsu K. Mitochondrial DNA as an inflammatory mediator in cardiovascular diseases. Biochem J. (2018) 475(5):839–52. 10.1042/BCJ20170714 29511093 PMC5840331

[46] Cheng X Zhao H Wen X Li G Guo S Zhang D. NLRP3-inflammasome Inhibition by MCC950 attenuates cardiac and pulmonary artery remodelling in heart failure with preserved ejection fraction. Life Sci. (2023) 333:122185. 10.1016/j.lfs.2023.122185 37858713

[47] Scandalis L Kitzman DW Nicklas BJ Lyles M Brubaker P Nelson MB. Skeletal muscle mitochondrial respiration and exercise intolerance in patients with heart failure with preserved ejection fraction. JAMA Cardiol. (2023) 8(6):575–84. 10.1001/jamacardio.2023.0957 37163294 PMC10173105

[48] Molina AJ Bharadwaj MS Van Horn C Nicklas BJ Lyles MF Eggebeen J. Skeletal muscle mitochondrial content, oxidative capacity, and Mfn2 expression are reduced in older patients with heart failure and preserved ejection fraction and are related to exercise intolerance. JACC Heart Fail. (2016) 4(8):636–45. 10.1016/j.jchf.2016.03.011 27179829 PMC4967040

[49] Bekfani T Pellicori P Morris DA Ebner N Valentova M Steinbeck L. Sarcopenia in patients with heart failure with preserved ejection fraction: impact on muscle strength, exercise capacity and quality of life. Int J Cardiol. (2016) 222:41–6. 10.1016/j.ijcard.2016.07.135 27454614

[50] Anker SD Butler J Filippatos G Ferreira JP Bocchi E Böhm M. EMPEROR-Preserved Trial investigators. Empagliflozin in heart failure with a preserved ejection fraction. N Engl J Med. (2021) 385(16):1451–61. 10.1056/NEJMoa2107038 34449189

[51] Solomon SD McMurray JJV Claggett B de Boer RA DeMets D Hernandez AF Inzucchi SE Kosiborod MN Lam CSP Martinez F Shah SJ. Dapagliflozin in heart failure with mildly reduced or preserved ejection fraction. N Engl J Med. 2022;387(12):1089–98. 10.1056/NEJMoa2206286 36027570

[52] Vaduganathan M Docherty KF Claggett BL Jhund PS de Boer RA Hernandez AF. SGLT-2 inhibitors in patients with heart failure: a comprehensive meta-analysis of five randomised controlled trials. Lancet. (2022) 400(10354):757–67. 10.1016/S0140-6736(22)01429-5; Epub 2022 August 27. Erratum in: Lancet. 2023 January 14;401(10371):104. doi: 10.1016/S0140-6736(23)00018-1. 36041474

[53] McDonagh TA Metra M Adamo M Gardner RS Baumbach A Böhm M. 2023 Focused update of the 2021 ESC guidelines for the diagnosis and treatment of acute and chronic heart failure. Eur Heart J. (2023) 44(37):3627–39. 10.1093/eurheartj/ehad195; Erratum in: Eur Heart J. 2024 January 1;45(1):53. doi: 10.1093/eurheartj/ehad613. 37622666

[54] Heidenreich PA Bozkurt B Aguilar D Allen LA Byun JJ Colvin MM. 2022 AHA/ACC/HFSA guideline for the management of heart failure: a report of the American College of Cardiology/American Heart Association joint committee on clinical practice guidelines. Circulation. (2022) 145(18):e895–e1032. 10.1161/CIR.0000000000001063; Epub 2022 April 1. Erratum in: Circulation. 2022 May 3;145(18):e1033. doi: 10.1161/CIR.0000000000001073. Erratum in: Circulation. 2022 September 27;146(13):e185. doi: 10.1161/CIR.0000000000001097. Erratum in: Circulation. 2023 April 4;147(14):e674. doi: 10.1161/CIR.0000000000001142. 35363499

[55] Kosiborod MN Abildstrøm SZ Borlaug BA Butler J Rasmussen S Davies M. Semaglutide in patients with heart failure with preserved ejection fraction and obesity. N Engl J Med. (2023) 389(12):1069–84. 10.1056/NEJMoa2306963 37622681

[56] Kosiborod MN Petrie MC Borlaug BA Butler J Davies MJ Hovingh GK. Semaglutide in patients with obesity-related heart failure and type 2 diabetes. N Engl J Med. (2024) 390(15):1394–407. 10.1056/NEJMoa2313917 38587233

[57] Packer M Zile MR Kramer CM Baum SJ Litwin SE Menon V. Tirzepatide for heart failure with preserved ejection fraction and obesity. N Engl J Med. (2025) 392(5):427–37. 10.1056/NEJMoa2410027 39555826

[58] European Medicines Agency. Outcome of assessment on use of Mounjaro in treatment of heart failure with preserved ejection fraction in adults with obesity. Amsterdam: European Medicines Agency; 30 January 2026. EMA/18704/2026; EMEA/H/C/005620/II/38. DOI: not applicable. Journal: not applicable.

[59] Greene SJ Fonarow GC Vaduganathan M Khan SS Butler J Gheorghiade M. The vulnerable phase after hospitalization for heart failure. Nat Rev Cardiol. (2015) 12(4):220–9. 10.1038/nrcardio.2015.14 25666406

[60] Spertus JA Jones PG Sandhu AT Arnold SV. Interpreting the Kansas city cardiomyopathy questionnaire in clinical trials and clinical care: JACC state-of-the-art review. J Am Coll Cardiol. (2020) 76(20):2379–90. 10.1016/j.jacc.2020.09.542 33183512

[61] Vaduganathan M Cunningham JW Claggett BL Causland FM Barkoudah E Finn P. Worsening heart failure episodes outside a hospital setting in heart failure with preserved ejection fraction: the PARAGON-HF trial. JACC Heart Fail. (2021) 9(5):374–82. 10.1016/j.jchf.2021.01.014 33839075

[62] Rommel KP von Roeder M Latuscynski K Oberueck C Blazek S Fengler K. Extracellular volume fraction for characterization of patients with heart failure and preserved ejection fraction. J Am Coll Cardiol. (2016) 67(15):1815–25. 10.1016/j.jacc.2016.02.018 27081022

[63] Taqueti VR Solomon SD Shah AM Desai AS Groarke JD Osborne MT. Coronary microvascular dysfunction and future risk of heart failure with preserved ejection fraction. Eur Heart J. (2018) 39(10):840–9. 10.1093/eurheartj/ehx721 29293969 PMC5939665

[64] Shah SJ Borlaug BA Kitzman DW McCulloch AD Blaxall BC Agarwal R. Research priorities for heart failure with preserved ejection fraction: national heart, lung, and blood institute working group summary. Circulation. (2020) 141(12):1001–26. 10.1161/CIRCULATIONAHA.119.041886 32202936 PMC7101072

[65] Mendieta G Ridker PM Borlaug BA Ducharme A Lam CSP Voors AA. Ziltivekimab in heart failure with preserved and mildly reduced ejection fraction: rationale and design of the ATHENA and HERMES trials. Eur J Heart Fail. (2026):xuag153. 10.1093/ejhf/xuag153 42106986

[66] Vardeny O Vaduganathan M Claggett BL Desai AS Jhund PS Lam CSP. Finerenone, Serum potassium, and clinical outcomes in heart failure with mildly reduced or preserved ejection fraction. JAMA Cardiol. (2025) 10(1):42–8. 10.1001/jamacardio.2024.4539 39550716 PMC11571067

