# Supplementary material for: Finerenone after FINEARTS-HF: evidence boundaries and implementation in heart failure with LVEF ≥40%
Source: Front Cardiovasc Med. 2026 Jun 16;13:1880045. doi: 10.3389/fcvm.2026.1880045 (PMC13314848; doi:10.3389/fcvm.2026.1880045)
Supplement: Supplementary file 1 [file Table1.pdf]

**Supplementary Table 1. Mechanistic evidence tiers for CKM-MR biology in HF with LVEF ≥40%**

| Pathway | Human signal | Finerenone-specific signal | Current boundary |
|---|---|---|---|
| Renal sodium avidity and potassium vulnerability | CKD, UACR elevation, congestion, and diuretic requirement identify higher-risk kidney-vascular biology. | FINEARTS-HF showed outcome benefit, early UACR reduction, and expected potassium/eGFR trade-offs. | UACR is useful for risk and pharmacodynamic assessment; mediation of HF benefit is unproven. |
| Endothelial inflammation and microvascular dysfunction | HFpEF is linked to inflammatory endothelial activation and coronary microvascular dysfunction. | Biologically plausible for MR blockade. | Use for mechanistic substudies, not routine response selection. |
| Fibrosis and extracellular matrix turnover | Myocardial stiffness, extracellular volume, and fibrosis markers relate to HFpEF biology and prognosis. | Antifibrotic rationale exists. | Do not claim proven fibrosis reversal as the mechanism of clinical benefit. |
| Mitochondrial and inflammasome signaling | Translational evidence implicates mitochondrial stress and inflammatory cascades in HFpEF and cardiac stress. | Direct human finerenone-response evidence is limited. | Hypothesis-generating only. |
| Body composition and skeletal muscle | Visceral adiposity, sarcopenia, myosteatosis, and impaired skeletal-muscle energetics influence symptoms and exercise | Obesity analyses support consistency, not validated response modification. | Avoid BMI-only phenotyping; test functional and body-composition endpoints prospectively. |

capacity.
